# Predictive accuracy of physicians’ estimates of outcome after severe stroke

**DOI:** 10.1371/journal.pone.0184894

**Published:** 2017-09-29

**Authors:** Marjolein Geurts, Floor A. S. de Kort, Paul L. M. de Kort, Julia H. van Tuijl, L. Jaap Kappelle, H. Bart van der Worp

**Affiliations:** 1 Department of Neurology and Neurosurgery, Brain Center Rudolf Magnus, University Medical Center Utrecht, Utrecht, the Netherlands; 2 Department of Neurology, Elisabeth-Twee Steden ziekenhuis, Tilburg, the Netherlands; University of Glasgow, UNITED KINGDOM

## Abstract

**Introduction:**

End-of-life decisions after stroke should be guided by accurate estimates of the patient’s prognosis. We assessed the accuracy of physicians’ estimates regarding mortality, functional outcome, and quality of life in patients with severe stroke.

**Methods:**

Treating physicians predicted mortality, functional outcome (modified Rankin scale (mRS)), and quality of life (visual analogue scale (VAS)) at six months in patients with major disabling stroke who had a Barthel Index ≤6 (of 20) at day four. Unfavorable functional outcome was defined as mRS >3, non-satisfactory quality of life as VAS <60. Patients were followed-up at six months after stroke. We compared physicians’ estimates with actual outcomes.

**Results:**

Sixty patients were included, with a mean age of 72 years. Of fifteen patients who were predicted to die, one actually survived at six months (positive predictive value (PPV), 0.93; 95% CI, 0.66–0.99). Of thirty patients who survived, one was predicted to die (false positive rate (FPR), 0.03; 95%CI 0.00–0.20). Of forty-six patients who were predicted to have an unfavorable outcome, four had a favorable outcome (PPV, 0.93; 95% CI, 0.81–0.98; FPR, 0.30; 95% CI; 0.08–0.65). Prediction of non-satisfactory quality of life was less accurate (PPV, 0.63; 95% CI, 0.26–0.90; FPR, 0.18; 95% CI 0.05–0.44).

**Conclusions:**

In patients with severe stroke, treating physicians’ estimation of the risk of mortality or unfavorable functional outcome at six months is relatively inaccurate. Prediction of quality of life is even more imprecise.

## Introduction

More than half of the patients with acute stroke are dead or disabled after two years [1]. In US studies, most in-hospital deaths of these patients occurred after a decision to withhold or withdraw life-sustaining therapies [2, 3]. These decisions usually evolve from complex discussions, in which accurate predictions of prognosis are crucial.

A wide range of prognostic models have been developed to aid prognostication after stroke, but none of these models is sufficiently accurate in the prediction of mortality or poor functional outcome to serve as the sole basis of decisions to limit treatment [4]. In addition, the large majority of these models are based on factors collected in the first hours after stroke onset, whereas treatment restrictions are often considered when there is no meaningful improvement during the first days or weeks [4]. Prognostication based on a physician’s estimate rather than on prognostic models can take into account factors that are usually not included in prognostic models, such as complications of stroke, previous comorbidities, changes in functional status over the course of hospitalization and estimated quality of life. However, the accuracy of prognostic estimates regarding mortality, functional outcome, and quality of life is uncertain. If the accuracy of physicians’ estimates is poor, physicians should be aware of prognostic uncertainties and their consequences when discussing end-of-life decisions.

In this study, we assessed the accuracy of treating physicians’ estimates in predicting mortality, functional outcome, and quality of life at six months in patients with severe disability at four days after stroke, and interpret these findings in the context of the end-of-life decision making process.

## Material and methods

### Patient selection

We studied patients included in the Advance Directive And Proxy opinions in acute sTroke (ADAPT) cohort, a prospective, two-center cohort study [5]. Consecutive patients admitted at the stroke unit with major disability, defined as Barthel Index (BI) ≤6 (out of 20) [6] at day four after ischemic stroke or intracerebral hemorrhage were eligible for participation. We restricted ourselves to this population because these are the patients in whom treatment restrictions are most often considered. Patients were included as soon as possible from four days after stroke and could be included until discharge.

Patients with a subarachnoid hemorrhage and patients without an available legal representative were excluded from the study. Patients were included between September 2012 and December 2013 in the University Medical Center Utrecht, a tertiary referral hospital, and between January and December 2013 in the St. Elisabeth hospital in Tilburg, a large regional teaching hospital, both in The Netherlands.

The study was approved by the institutional review board of the University Medical Center Utrecht and of the St. Elisabeth hospital. Written informed consent was obtained from each patient or a legal representative.

### Data collection

We collected information on patient characteristics (age, sex), type of stroke (ischemic stroke or intracerebral hemorrhage), stroke severity on admission (by means of National Institutes of Health Stroke Scale (NIHSS)) [7] and pre-stroke comorbidity with use of the Charlson Comorbidity Index (CCI) [8].

### Physicians’ estimates

The treating physicians were neurology residents assigned to the daily care of patients during admission. These residents were supervised in the daily care of patients by experienced stroke neurologists. Prognosis and the need of installment of end-of-life decisions were discussed by the neurologist and the resident on a daily basis. The neurology resident predicted outcome after six months immediately after patient inclusion by a questionnaire regarding the prediction of mortality, functional outcome (as measured with the modified Rankin Scale (mRS)), [9] and quality of life (as measured with a visual analogue scale (VAS)) [10]. Scores on the mRS range from 0 (no symptoms) through to 5 (severe disability); for statistical purposes, death was given a score of 6. The VAS was a vertical line of 10 centimeters with a ‘☺’ at the top demarcating the best possible quality of life and a ‘☹’ at the lower end for the worst possible quality of life. Scores were calculated as the indicated level in (centimeters/10)*100. Quality of life was considered acceptable if VAS ≥60 [11] No formal prediction models were used in the daily care of the patients, nor in the estimation of outcomes.

### Follow-up

A single trained investigator (FASdK), blinded to the physicians’ predictions, visited each patient and caregiver at six months (+/- six weeks) after stroke to assess functional outcome (as measured with the mRS and BI) and quality of life (as measured with a VAS and with the Medical Outcomes Study 36-item short-form health survey (SF-36)). For the SF-36, two summary scores were calculated as a representation of physical or mental health [12].

### Statistical analyses

The primary outcomes were the physicians’ accuracies regarding the prediction of mortality, functional outcome, and quality of life at six months. Predictions of functional outcome were considered correct if the prediction of either favorable (mRS 0–3) or unfavorable (mRS 4–6) functional outcome was correct. Prediction of quality of life was considered correct if the prediction of satisfactory quality of life (VAS ≥60) or non-satisfactory quality of life (VAS <60) was correct.

In a secondary analysis, prediction of functional outcome was considered correct if there was an exact agreement on the mRS.

Accuracy results for mortality and the dichotomized mRS and VAS outcomes were measured by calculating the positive predictive values (PPV), negative predictive values (NPV), and false positive rate (FPR) with corresponding 95% confidence intervals (CI). We present PPV, NPV and FPR as measures of test performance (rather than sensitivity and specificity), because these tests will provide the clinically most important results, taking into account the context of end-of-life decisions they are used in. An incorrect prediction of a poor outcome can have the irreversible consequence of withdrawing or withholding life-sustaining therapies. In this context, we are interested in how likely it is that if the physician predicts a poor outcome (the test is positive), the patient really has a poor outcome (and the installment of a treatment restriction can be justified). This is represented by the positive predictive value. In other words: a high positive predictive value means you can trust the physicians’ prediction it if it is positive. The same is true for a predicted good outcome (the test is negative), which is represented by the negative predictive value.

We used χ^2^-test to compare PPVs between groups.

As a cut-off point for predictive accuracy, we used a false positive rate for predicted mortality, poor functional outcome or unsatisfactory quality of life of 0.01.

#### Sample size calculation

To achieve a precision (the maximum difference between estimated FPR and the true value) of 10% for FPR, using an expected FPR of 0.05 and an expected prevalence of 0.50 for either mortality, poor functional outcome or unsatisfactory quality of life, we need a total sample size of 39 patients.

#### Subgroup analyses

Predefined subgroup analyses were done with regard to type of stroke and in patients who had no treatment restrictions, including no do-not-resuscitate (DNR) orders. The relation between treatment restrictions and predicted outcomes was calculated with Poisson regression analysis with a robust error, and expressed as relative risk (RR) with corresponding 95% CI.

## Results

We included 60 patients with a mean age of 72 years (SD, 15) and a median Barthel Index of 0 (range, 0–6). Thirty-six (60%) patients had an ischemic stroke. The median time from admission to inclusion was six days (range, 4–10). Baseline characteristics are presented in Table 1.

**Table 1:** Demographic characteristics of included patients.

	All patients *n* = 60
Age (years)	72 (15)
Men	30 (50)
Ischemic stroke	36 (60)
NIHSS on admission	16 (6)
CCI	1 (0–6)
Barthel Index at day 4	0 (0–6)

Data are n (%), median (range), or mean (standard deviation (SD)) where appropriate. CCI, comorbidity by the ICD-9-CM version of the Charlson Comorbidity Index; NIHSS, National Institutes of Health Stroke Scale.

Twenty-one neurology residents, supervised by 14 stroke neurologists filled out the questionnaires. The number of patients treated by one physician ranged from one to eight.

### Mortality

**Fig 1:**
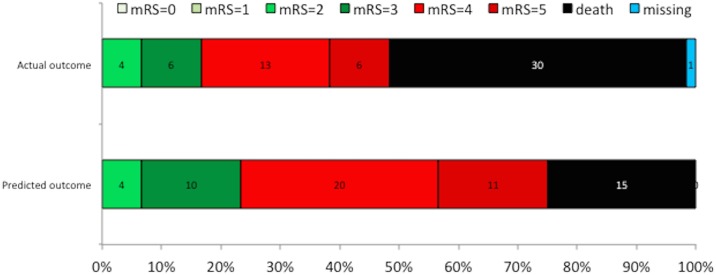
Distribution of predicted and actual modified Rankin Scale (mRS) grades at 6 months.

At six months, 30 patients (50%) had died. Of 30 surviving patients, one patient had been predicted to die (FPR, 0.03; 95%CI; 0.00–0.20). Directly after inclusion, physicians predicted 15 patients to die; fourteen of them actually died (PPV, 0.93; 95% CI, 0.66–0.99) (Table 2, Fig 1).

**Table 2:** Accuracy of prognostic estimates.

	Predicted outcome	Actual outcome		Predictive value	95% CI
**Mortality**		Death	Alive		
	Death	14	1	PPV 0.93	0.66–0.99
	Alive	16	29	NPV 0.64	0.49–0.78
				FPR 0.03	0.00–0.20
**Functional outcome**		Unfavorable	Favorable		
	Unfavorable	42	3	PPV 0.93	0.81–0.98
	Favorable	7	7	NPV 0.50	0.24–0.76
				FPR 0.30	0.08–0.65
**Quality of life**		Non-satisfactory	Satisfactory		
	Non-satisfactory	5	3	PPV 0.63	0.26–0.90
	Satisfactory	4	14	NPV 0.78	0.52–0.93
				FPR 0.18	0.05–0.44

CI, confidence interval; PPV, positive predictive value; NPV, negative predictive value; FPR, false positive rate

### Functional outcome

Functional outcome at six months could be assessed in 59 patients; one patient declined follow-up. Thirty patients died, of the survivors 19 (65%) had an unfavorable outcome. The median mRS in survivors was 4 (range, 2–5) (Table 3 and Fig 1).

**Table 3:** Outcomes at six months.

	All patients (n = 60)
Death	30 (50%)
Poor outcome (mRS 4–6)*	49 (83%)
Poor outcome in survivors (mRS 4–5)*	19 (65%)
Barthel Index*	11 (12)
Quality of life (VAS)†	60 (17)
Non-satisfactory QoL	9 (35%)
Quality of life (SF-36)‡	
Physical summary	19 (17)
Mental summary	83 (20)

Data are n (%), median (range), median (interquartile range (IQR)) or mean (standard deviation (SD)) where appropriate. mRS, modified Rankin scale; VAS, visual analogue scale; SF-36, short form 36 questionnaire.

*n = 29;

^†^n = 26;

^‡^n = 26

Of 45 patients who were predicted to have an unfavorable outcome (dichotomized mRS), 42 had a unfavorable outcome (PPV, 0.93; 95% CI, 0.81–0.98). Of the 14 patients who were predicted to have a favorable outcome seven had a favorable outcome (NPV, 0.50; 95% CI, 0.24–0.76) (Table 2).

**Fig 2:**
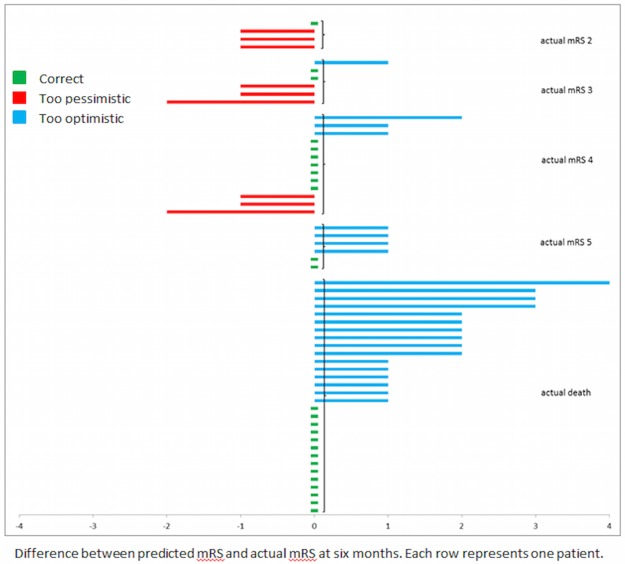
Predicted and observed 6 month functional outcome (mRS score, range 0–6) per patient (n = 59).

In 26 of 59 patients (44%), the exact (nominal) prediction of mRS score was correct. The majority (73%) of incorrect predictions on functional outcome were too optimistic (Fig 2).

### Quality of life

Data on quality of life were available in 26 of 30 surviving patients. Two patients declined follow-up on quality of life, one patient was moribund at the time of follow-up, one patient could not answer the questions because of severe aphasia. The results as measured with the SF-36 are presented in Table 3. Seventeen (61%) of the 26 survivors had a satisfactory quality of life. The mean score on the VAS was 60 (SD 17). Of eight surviving patients who were predicted to have a non-satisfactory quality of life, 5 had a non-satisfactory quality of life at six months (PPV, 0.63; 95% CI, 0.26–0.90). Of the 18 surviving patients who were predicted to have a satisfactory quality of life, 14 actually had a satisfactory quality of life at six months (NPV, 0.78; 95% CI, 0.52–0.93) (Table 2).

### Subgroup analyses

There were no differences between patients with ischemic stroke and intracerebral hemorrhage concerning the predictive accuracy of mortality (P = 0.27), unfavorable functional outcome (P = 0.11), or unsatisfactory quality of life (P = 0.69) (S1 and S2 Tables).

18 of 60 patients had no treatment restrictions. In these patients, predictive accuracy of unfavorable functional outcome was essentially the same as in the total group (PPV, 0.88; 95%CI, 0.47–0.99), but prognostic errors were more optimistic (S3 Table).

The installment of treatment restrictions was strongly associated with a predicted unfavorable outcome and a predicted unsatisfactory quality of life (Relative Risk (RR), 11.9; 95%CI, 3.0–47.6; P<0.001 and RR, 8.8; 95%CI, 3.6–21.2; P<0.001 respectively).

## Discussion

This study shows that in patients with severely disabling stroke, defined as a Barthel Index ≤6 after four days, treating physicians’ estimation of the risk of death or unfavorable functional outcome at six months is relatively inaccurate. Prediction of quality of life is even more imprecise.

Accurate information about the expected outcome of disease is required to guide physicians and other professionals, patients, and their relatives in making decisions related to the withdrawal or withholding of life-sustaining treatments. If an expected negative outcome (death, unfavorable functional outcome or a non-satisfactory quality of life) is used as a basis for treatment restrictions, the predictive accuracy should be very high to prevent unfounded pessimism which can lead to early withdrawal of treatment in a patient that otherwise could have recovered. The false positive rate of a predicted poor outcome should preferably be zero, with a narrow confidence interval. At present, such predictive accuracy only exists for prognostic models in comatose patients after cardiopulmonary resuscitation for cardiac arrest [13] and not for stroke patients. In our opinion, the predictive accuracy of physicians is insufficient to serve as the sole basis of decisions to limit treatment. Physicians should be aware of prognostic uncertainties and their consequences when discussing end-of-life decisions.

In this study, physicians predicted unfavorable functional outcome better than a non-satisfactory quality of life, probably because quality of life is not only related to the severity of disability, but also to factors such as the presence or absence of meaningful activities and social or emotional support [14] which are often not identified during admission. Moreover, patients often report greater happiness and quality of life than healthy people predict they would feel under the same circumstances, a phenomenon often referred to as a ‘disability paradox’, which is explained in part by the capacity of patients with chronic illness or disability to adapt to their circumstances [15].

The accuracy of the physician’s prognostic estimates in this study is in the same range as in a previous study where neurovascular fellows predicted functional outcome at six months in patients with subarachnoid hemorrhage [16] and in a study where junior neurointensivists predicted functional outcome and quality of life at six months in patients requiring mechanical ventilation in any neurological disease [17]. Previous studies compared the accuracy of prediction models to physicians’ estimates on mortality and functional outcome, and found the prediction models to be more accurate in patients with ischemic stroke, [18, 19], but not in patients with intracerebral hemorrhage [20].

The frequently used iScore for ischemic stroke and ICH score for intracerebral hemorrhage have areas under the curve for case fatality at 30 days of 0.79 and 0.88, respectively, in validation studies [21, 22]. This means that both prognostic models and physicians’ prognostic estimates lack the accuracy to serve as sole base for end-of-life decisions. The predictive accuracy might increase when using a combination of ‘mathematical’ prediction models and physicians’ prognostic estimates, but this requires further research.

This study has limitations. First, we included both patients with ischaemic and hemorrhagic stroke. These entities are inherently different with respect to prognosis. Second, our findings cannot be generalized to an unselected population of stroke patients. We included patients who were alive but severely dependent on day four after stroke, because treatment restrictions are probably most often installed in this patient group. As a result of this selection, our results apply only to this selected group of patients and cannot be extrapolated to patients in the first days after stroke, those who are less severely disabled at day four after stroke or to patients with other diseases. Quality of life predictions were assessed in an even more selected group, because only survivors could be evaluated. The number of patients available to assess predictive accuracy of quality of life lack the statistical power to draw firm conclusions. Third, quality of life data should be interpreted with caution because patients could have given desired answers during the home visit. Fourth, the cut-off value for predictive accuracy is rather arbitrary and represents the authors’ interpretation based on previously presented visions [4]. The lower the false positive rate, the more decisions to withhold or withdraw life-sustaining therapies are based on correct predictions of poor outcome. Fifth, self-fulfilling prophecies are a major concern when assessing prognostic accuracy. In the present study, patients with a predicted poor prognosis more frequently had treatment restrictions, which have been associated with an actual poor outcome in previous studies [2, 5, 23, 24]. Only 18 patients received full supportive care, a number too small to draw firm conclusions. Finally, improvement of functional outcome and quality of life may still occur after six months [11] and our results therefore do not represent a completed recovery.

In patients with severe stroke, treating physicians’ estimation of the risk of mortality or unfavorable functional outcome at six months is relatively inaccurate. Prediction of quality of life is even more imprecise.

Future research should focus on how to improve predictive accuracy, for example by using a combination of ‘mathematical’ prediction models and physicians’ prognostic estimates, and on how to identify in the acute stage after stroke patients who will recapture a good quality of life despite poor functional outcome.

## Supporting information

S1 Table Outcome measures in subgroup analysis patients with ischemic stroke. (DOCX)

S2 Table Outcome measures in subgroup analysis patients with intracerebral hemorrhage. (DOCX)

S3 Table Outcome measures in subgroup analysis patients without treatment restrictions. (DOCX)

## References

[1] Luengo-Fernandez R, Paul NL, Gray AM, Pendlebury ST, Bull LM, Welch SJ, et al Population-based study of disability and institutionalization after transient ischemic attack and stroke: 10-year results of the Oxford Vascular Study. Stroke 2013;44:2854–61 doi: 10.1161/STROKEAHA.113.001584 23920019 10.1161/STROKEAHA.113.001584 PMC4946627

[2] Becker KJ, Baxter AB, Cohen WA, Bybee HM, Tirschwell DL, Newell DW, et al Withdrawal of support in intracerebral hemorrhage may lead to self-fulfilling prophecies. Neurology 2001;56:766–72. 11274312 10.1212/wnl.56.6.766

[3] Kelly AG, Hoskins KD, Holloway RG. Early stroke mortality, patient preferences, and the withdrawal of care bias. Neurology 2012;79:941–4 doi: 10.1212/WNL.0b013e318266fc40 22927679 10.1212/WNL.0b013e318266fc40 PMC3425847

[4] Geurts M, Macleod MR, van Thiel GJ, van Gijn J, Kappelle LJ, van der Worp HB. End-of-life decisions in patients with severe acute brain injury. Lancet Neurol 2014;13:515–24 doi: 10.1016/S1474-4422(14)70030-4 24675048 10.1016/S1474-4422(14)70030-4

[5] Geurts M, De Kort FAS, De Kort PLM, van Tuijl JH, van Thiel GJMW, Kappelle LJ, et al Treatment restrictions in patients with severe stroke are associated with an increased risk of death. European Stroke Journal. doi: 10.1177/2396987317704546 10.1177/2396987317704546 PMC5992732 29900408

[6] Mahoney FI, Barthel DW. Functional Evaluation: the Barthel Index. Md State Med J 1965;14:61–5. 14258950

[7] Brott T, Adams HP Jr, Olinger CP, Marler JR, Barsan WG, Biller J, et al Measurements of acute cerebral infarction: a clinical examination scale. Stroke 1989;20:864–70. 2749846 10.1161/01.str.20.7.864

[8] Charlson ME, Pompei P, Ales KL, MacKenzie CR. A new method of classifying prognostic comorbidity in longitudinal studies: development and validation. J Chronic Dis 1987;40:373–83. 3558716 10.1016/0021-9681(87)90171-8

[9] van Swieten JC, Koudstaal PJ, Visser MC, Schouten HJ, van Gijn J. Interobserver agreement for the assessment of handicap in stroke patients. Stroke 1988;19:604–7. 3363593 10.1161/01.str.19.5.604

[10] Indredavik B, Bakke F, Slordahl SA, Rokseth R, Håheim LL. Stroke unit treatment improves long-term quality of life: a randomized controlled trial. Stroke 1998;29:895–9. 9596231 10.1161/01.str.29.5.895

[11] Geurts M, van der Worp HB, Kappelle LJ, Amelink GJ, Algra A, Hofmeijer J, et al Surgical decompression for space-occupying cerebral infarction: outcomes at 3 years in the randomized HAMLET trial. Stroke 2013;44:2506–8 doi: 10.1161/STROKEAHA.113.002014 23868265 10.1161/STROKEAHA.113.002014

[12] Ware JE, Snow KK, Kosinski M, Gandek B., eds. SF-36 Health Survey: Manual and Interpretation Guide. Boston: he Health Institute, New England Medical Center 1993.

[13] Wijdicks EF, Hijdra A, Young GB, Bassetti CL, Wiebe S; Quality Standards Subcommittee of the American Academy of Neurology. Practice parameter: prediction of outcome in comatose survivors after cardiopulmonary resuscitation (an evidence-based review): report of the Quality Standards Subcommittee of the American Academy of Neurology. Neurology 2006;67:203–10 67/2/203 doi: 10.1212/01.wnl.0000227183.21314.cd 16864809 10.1212/01.wnl.0000227183.21314.cd

[14] Cerniauskaite M, Quintas R, Koutsogeorgou E, Meucci P, Sattin D, Leonardi M, et al Quality-of-life and disability in patients with stroke. Am J Phys Med Rehabil 2012;91:S39–47 doi: 10.1097/PHM.0b013e31823d4df7 22193309 10.1097/PHM.0b013e31823d4df7

[15] Ubel PA, Loewenstein G, Schwarz N, Smith D. Misimagining the unimaginable: the disability paradox and health care decision making. Health Psychol 2005;24:S57–62 2005-08085-009 doi: 10.1037/0278-6133.24.4.S57 16045420 10.1037/0278-6133.24.4.S57

[16] Navi BB, Kamel H, McCulloch CE, Nakagawa K, Naravetla B, Moheet AM, et al Accuracy of neurovascular fellows' prognostication of outcome after subarachnoid hemorrhage. Stroke 2012;43:702–7 doi: 10.1161/STROKEAHA.111.639161 22223238 10.1161/STROKEAHA.111.639161

[17] Finley Caulfield A, Gabler L, Lansberg MG, Eyngorn I, Mlynash M, Buckwalter MS, et al Outcome prediction in mechanically ventilated neurologic patients by junior neurointensivists. Neurology 2010;74:1096–101 doi: 10.1212/WNL.0b013e3181d8197f 20368630 10.1212/WNL.0b013e3181d8197f PMC2865775

[18] Weimar C, Konig IR, Kraywinkel K, Ziegler A, Diener HC; German Stroke Study Collaboration. Age and National Institutes of Health Stroke Scale Score within 6 hours after onset are accurate predictors of outcome after cerebral ischemia: development and external validation of prognostic models. Stroke 2004;35:158–62 doi: 10.1161/01.STR.0000106761.94985.8B 14684776 10.1161/01.STR.0000106761.94985.8B

[19] Saposnik G, Cote R, Mamdani M, Raptis S, Thorpe KE, Fang J, et al JURaSSiC: accuracy of clinician vs risk score prediction of ischemic stroke outcomes. Neurology 2013;81:448–55 doi: 10.1212/WNL.0b013e31829d874e 23897872 10.1212/WNL.0b013e31829d874e PMC3776534

[20] Hwang DY, Dell CA, Sparks MJ, Watson TD, Langefeld CD, Comeau ME, et al Clinician judgment vs formal scales for predicting intracerebral hemorrhage outcomes. Neurology 2016;86:126–33 doi: 10.1212/WNL.0000000000002266 26674335 10.1212/WNL.0000000000002266 PMC4731687

[21] Saposnik G, Kapral MK, Liu Y, Hall R, O'Donnell M, Raptis S, et al IScore: a risk score to predict death early after hospitalization for an acute ischemic stroke. Circulation 2011;123:739–49 doi: 10.1161/CIRCULATIONAHA.110.983353 21300951 10.1161/CIRCULATIONAHA.110.983353

[22] Clarke JL, Johnston SC, Farrant M, Bernstein R, Tong D, Hemphill JC 3rd. External validation of the ICH score. Neurocrit Care 2004;1:53–60 NCC:1:1:53 doi: 10.1385/NCC:1:1:53 16174898 10.1385/NCC:1:1:53

[23] Hemphill JC 3rd, Newman J, Zhao S, Johnston SC. Hospital usage of early do-not-resuscitate orders and outcome after intracerebral hemorrhage. Stroke 2004;35:1130–4 doi: 10.1161/01.STR.0000125858.71051.ca 15044768 10.1161/01.STR.0000125858.71051.ca

[24] Silvennoinen K, Meretoja A, Strbian D, Putaala J, Kaste M, Tatlisumak T. Do-not-resuscitate (DNR) orders in patients with intracerebral hemorrhage. Int J Stroke 2014;9:53–8 doi: 10.1111/ijs.12161 24148872 10.1111/ijs.12161

