# Supplementary material for: Predictive accuracy of physicians’ estimates of outcome after severe stroke
Source: PLoS One. 2017 Sep 29;12(9):e0184894. doi: 10.1371/journal.pone.0184894 (PMC5621670; doi:10.1371/journal.pone.0184894)
Supplement: S2 Table — (DOCX) [file pone.0184894.s002.docx]

S2 Table. Outcome measures in subgroup analysis patients with intracerebral hemorrhage

|  | Predicted outcome | Actual outcome | | **Predictive value** | **95% CI** |
| --- | --- | --- | --- | --- | --- |
| **Mortality** |  | Death | Alive |  |  |
|  | Death | 8 | 0 | PPV 1.00 | 0.60-1.00 |
|  | Alive | 7 | 9 | NPV 0.56 | 0.31-0.79 |
|  |  |  |  | FPR 0.00 | 0.00-0.37 |
| **Functional outcome** |  | Unfavorable | Favorable |  |  |
|  | Unfavorable | 17 | 0 | PPV 1.00 | 0.77-1.00 |
|  | Favorable | 4 | 3 | NPV 0.43 | 0.12-0.80 |
| **Quality of life** |  | Non-satisfactory | Satisfactory | FPR 0.00 | 0.00-0.69 |
|  | Non-satisfactory | 1 | 2 | PPV 0.33 | 0.09-0.69 |
|  | Satisfactory | 1 | 5 | NPV 0.83 | 0.36-0.99 |
|  |  |  |  | FPR 0.29 | 0.05-0.70 |
| CI, confidence interval; PPV, positive predictive value; NPV, negative predictive value; FPR, false positive rate | | | | | |
