# Supplementary material for: Predictive accuracy of physicians’ estimates of outcome after severe stroke
Source: PLoS One. 2017 Sep 29;12(9):e0184894. doi: 10.1371/journal.pone.0184894 (PMC5621670; doi:10.1371/journal.pone.0184894)
Supplement: S3 Table — (DOCX) [file pone.0184894.s003.docx]

S3 Table. Outcome measures in subgroup analysis patients without treatment restrictions

|  | Predicted outcome | Actual outcome | | **Predictive value** | **95% CI** |
| --- | --- | --- | --- | --- | --- |
| **Mortality** |  | Death | Alive |  |  |
|  | Death | 0 | 0 | NA | NA |
|  | Alive | 2 | 16 | NPV 0.89 | 0.64-0.98 |
|  |  |  |  | FPR 1.00 | 0.20-1.00 |
| **Functional outcome** |  | Unfavorable | Favorable |  |  |
|  | Unfavorable | 7 | 1 | PPV 0.88 | 0.47-0.99 |
|  | Favorable | 4 | 6 | NPV 0.60 | 0.27-0.86 |
| **Quality of life** |  | Non-satisfactory | Satisfactory | FPR 0.14 | 0.01-0.58 |
|  | Non-satisfactory | 3 | 1 | PPV 0.75 | 0.22-0.99 |
|  | Satisfactory | 3 | 9 | NPV 0.75 | 0.43-0.93 |
|  |  |  |  | FPR 0.10 | 0.01-0.46 |
| CI, confidence interval; PPV, positive predictive value; NPV, negative predictive value; FPR, false positive rate | | | | | |
